# Small-molecule Wnt inhibitors are a potential novel therapy for intestinal fibrosis in Crohns disease

**DOI:** 10.1042/CS20210889

**Published:** 2022-10-14

**Authors:** Amy Lewis, Saray Sánchez, Giulio Berti, Belen Pan-Castillo, Anke Nijhuis, Shameer Mehta, Liliane Eleid, Hannah Gordon, Radha Gadhok, Christopher Kimberley, Annamaria Minicozzi, Joanne Chin-Aleong, Roger Feakins, Robert Kypta, James Oliver Lindsay, Andrew Silver

**Affiliations:** 1 Centre for Genomics and Child Health, Blizard Institute, Barts and The London School of Medicine & Dentistry, London E1 2AT, U.K.; 2 Cancer Heterogeneity Lab, CIC bioGUNE, 48160 Derio, Spain; 3 Centre for Immunobiology, Blizard Institute, Barts and The London School of Medicine & Dentistry, London E1 2AT, U.K.; 4 Department of Colorectal Surgery, Division of Surgery & Perioperative Care, The Royal London Hospital, Whitechapel, London E1 1BB, U.K.; 5 Department of Histopathology, The Royal London Hospital, London E1 1BB, U.K.; 6 Department of Cellular Pathology, Royal Free London NHS Foundation Trust, London NW3 2QG, U.K.; 7 Department of Surgery and Cancer, Imperial College London, London W12 0NN, U.K.

**Keywords:** Crohns disease, fibrosis, inhibitors, intestine, Wnt proteins

## Abstract

Intestinal fibrosis and stricture formation is an aggressive complication of Crohns disease (CD), linked to increased morbidity and costs. The present study investigates the contribution of Wingless-Int-1 (Wnt) signalling to intestinal fibrogenesis, considers potential cross-talk between Wnt and transforming growth factor β1 (TGFβ) signalling pathways, and assesses the therapeutic potential of small-molecule Wnt inhibitors.

β-catenin expression was explored by immunohistochemistry (IHC) in formalin-fixed paraffin embedded (FFPE) tissue from patient-matched nonstrictured (NSCD) and strictured (SCD) intestine (*n*=6 pairs). Functional interactions between Wnt activation, TGFβ signalling, and type I collagen (Collagen-I) expression were explored in CCD-18Co cells and primary CD myofibroblast cultures established from surgical resection specimens (*n*=16) using small-molecule Wnt inhibitors and molecular techniques, including siRNA-mediated gene knockdown, immunofluorescence (IF), Wnt gene expression arrays, and western blotting. Fibrotic SCD tissue was marked by an increase in β-catenin-positive cells. *In vitro*, activation of Wnt-β-catenin signalling increased Collagen-I expression in CCD-18Co cells. Conversely, ICG-001, an inhibitor of β-catenin signalling, reduced Collagen-I expression in cell lines and primary CD myofibroblasts. TGFβ increased β-catenin protein levels but did not activate canonical Wnt signalling. Rather, TGFβ up-regulated WNT5B, a noncanonical Wnt ligand, and the Wnt receptor FZD8, which contributed directly to the up-regulation of Collagen-I through a β-catenin-independent mechanism. Treatment of CCD-18Co fibroblasts and patient-derived myofibroblasts with the FZD8 inhibitor 3235-0367 reduced extracellular matrix (ECM) expression. Our data highlight small-molecule Wnt inhibitors of both canonical and noncanonical Wnt signalling, as potential antifibrotic drugs to treat SCD intestinal fibrosis. They also highlight the importance of the cross-talk between Wnt and TGFβ signalling pathways in CD intestinal fibrosis.

## Introduction

Intestinal fibrosis with stricture formation and associated bowel obstruction is a common and serious complication of Crohns disease (CD) [1]. Strictures occur in over a third of CD patients and, because of an absence of specific antifibrotic medical therapies, are the main indication for surgery [2]. Surgery is noncurative, recurrence is common, and current medical therapies such as anti-TNFs appear not to reduce fibrosis or the need for stricture resection [3,4]. Consequently, stricturing CD (SCD) is associated with high direct and indirect healthcare costs, reduced quality of life, and short bowel syndrome caused by repeat surgeries [5]. Targeted therapies based on a better understanding of the underlying molecular mechanisms of intestinal fibrosis are needed urgently.

Intestinal fibrosis is part of a complex process that involves all layers of the intestine, multiple cell-types and signalling pathways, as well as interactions between genetic susceptibility loci and environmental triggers. Inflammation is an important initial driver of fibrosis, but there is growing evidence that overtime fibrosis can progress independently from inflammation. A key feature of intestinal fibrosis is an increase in activated myofibroblasts, which are the main source of increased production of extracellular matrix (ECM) proteins, e.g. type I collagen (Collagen-I). In CD patients, fibroblast activation has been linked to increased expression of transforming growth factor β1 (TGFβ) and activation of SMAD signalling [6,7]. However, targeting the TGFβ pathway to treat fibrosis might interfere with the important role played by TGFβ in constraining inflammation in CD [8].

Cross-talk between TGFβ and Wingless-Int-1 (Wnt) signalling has been reported widely [9]. Both pathways appear important for normal wound healing and sustained fibroblast activation during fibrosis [10,11]. In a pathological context, Wnt activation is linked to fibrosis in multiple organs including the lung, liver, kidney, and skin [12]. Initiation of Wnt signalling requires binding of Wnt ligands to transmembrane frizzled receptors (FZD) and coreceptors, such as LRP5/6 and ROR1/2, which can activate canonical or noncanonical pathways, depending on coreceptor and cell context [13]. In the canonical (Wnt/β-catenin) pathway, Wnt ligand binding initiates changes that lead to the inhibition of the β-catenin destruction complex, resulting in β-catenin accumulation in the cytosol and translocation into the nucleus. Here, β-catenin forms transcriptional complexes with cofactors, such as CREB-binding protein (CBP), and transcription factors, including T-cell factor/lymphoid enhancer factor (TCF/LEF) family, to promote the expression of target genes that control cell fate and proliferation [11]. In contrast, noncanonical Wnt signalling is β-catenin-independent and can be subdivided into the planar cell polarity (PCP) and the Wnt/Ca2+ pathways.

The mechanisms by which Wnt signalling promotes fibrosis are complex, cell-type, and context-dependent; and both canonical and noncanonical pathways have been implicated. For example, in many organs, fibrosis is often associated with increased nuclear β-catenin [14–18], and accompanied by increased expression of Wnt ligands, FZD receptors, and Wnt target gene expression [18–20]. Other studies have highlighted the role of noncanonical Wnt-5A signalling via FZD8, in TGFβ-mediated fibrosis in airway smooth muscle cells, the liver, and the lungs [21–23]. Conversely, inhibitors of Wnt signalling limit fibrosis. For example, Wnt-C59, which inhibits porcupine to block Wnt secretion, attenuates renal and cardiac fibrosis [24,25]. Small-molecule inhibitors of FZD8 have been shown to inhibit ECM gene expression [26–28]. Moreover, ICG-001, which blocks β-catenin’s interaction with CBP [29], inhibits fibrosis in the lungs, kidneys, and liver [30–32], and has positive results in patients with hepatitis C virus-related cirrhosis [33]. ICG-001 can also uncouple β-catenin/CBP-dependent signalling downstream of TGF-β by disrupting interactions between Smad3 and the β-catenin/CBP-complex [34].

In the intestine, Wnt signalling is required to maintain epithelial homeostasis, promote Paneth cell differentiation, and maintain α-defensin production [35]. Stromal and mesenchymal cells are important sources of Wnt ligands, which contribute to structural remodelling of the intestine during inflammation [36,37]. In CD patients, single nucleotide polymorphisms have been linked to Wnt-responsive DNA enhancer elements and to increased expression of the Wnt-responsive gene c-MYC [38]. Moreover, Wnt activation has been linked to the development of aggressive complications in inflammatory bowel disease (IBD) patients, including IBD colorectal carcinogenesis [39], and development of intestinal fistulae [40]. Increased staining of nuclear β-catenin has also been reported in CD fibrosis [41]. However, human-based mechanistic studies and evaluation of Wnt inhibitors in cellular models of SCD have been lacking.

In the present study, we confirm reports of increased β-catenin in SCD and demonstrate a role of β-catenin in the regulation of Collagen-I in cell lines and primary CD myofibroblasts. We further explore cross-talk between the TGFβ and Wnt signalling in intestinal fibroblasts and highlight a novel role for FZD8 in SCD. Moreover, we evaluate the ability of the small-molecule Wnt signalling inhibitors to attenuate TGFβ-mediated intestinal fibrosis in models of intestinal fibrosis and primary CD fibroblast cultures. Our data provide important preclinical evidence for the potential use of small-molecule inhibitors of Wnt signalling as a potential novel therapy for SCD.

## Materials and methods

### Patient samples and ethics

The study was conducted in accordance with the latest version of the declaration of Helsinki. Appropriate local Ethics Committee approval was obtained from the London—City Road & Hampstead Research Ethics Committee (15/LO/2127) and Informed consent were obtained prior to patient recruitment.

### Immunohistochemistry

Formalin-fixed paraffin embedded (FFPE) surgical resection tissue from the ileum of CD patients with a documented stricture was obtained from the Barts Health NHS Trust histopathology archive. From each CD patient, SCD and nonstrictured CD (NSCD) FFPE blocks (*n*=6, pairs, Supplementary Table S1) were identified from pathology reports and the relative levels of inflammation and fibrosis assessed in detail by a pathologist (R.F.), as described previously [42]. All immunohistochemistry (IHC) for β-catenin (760-4242, Roche) was performed by Pathognomics Ltd (Huntingdon, UK), using diagnostic protocols optimised for the automated Ventana stains system (Roche). A pathologist (R.F.) reviewed all slides and the percentage of β-catenin-positive cells in the stroma and *muscularis propria* (MP) calculated. For IHC image analysis, six paired of SCD and NSCD tissues (*n*=6) were analysed for β-catenin. For quantification of the percentage of β-catenin cells, six fields of view per slide, three from the mucosa and three from MP, were selected. β-catenin-positive cells were then manually scored in a blinded fashion using the ImageJ point picker plugin. The numbers of β-catenin-positive cells were expressed as a percentage of the total number of cells in that field. In the mucosa, only stromal cells were scored, and morphologically identifiable epithelial and endothelial cells or nerve ganglions were excluded. Similarly, endothelial and nerve cells were also excluded from the scoring the MP. One NSCD block did not contain tissue from the MP; therefore, the paired analysis for the MP related to five SCD and NSCD paired tissues (*n*=5). Typically, 100–300 cells were scored per field of view.

### Cell culture and treatments

CCD-18Co (normal human intestinal fibroblasts, ATCC® CRL-1459™) were cultured according to guidelines. For all cell treatments, cells were serum starved overnight prior to 48 h treatment with 10 µM ICG-001 dissolved in DMSO, or 100 nM porcupine inhibitor Wnt-C59 (Selleckchem S7037), or 10 μM FZD8 inhibitor 3235-0367 (C1, ChemDiv), alone or in combination with 10 ng/mL-recombinant human TGFβ1 (R&D Systems). For FZD8 siRNA-mediated gene knockdowns in 96-well plates, cells were transfected with either a siRNA-targeted against FZD8 (25 nM, SC-39992) or a nontargeted control (SC-37007) using DharmaFECT 2 reagent (T-2002-02). Transfections were performed according to the Dharmacon™ DharmaFECT™ 1–4 transfection protocol, using 0.1 μL of DharmaFECT 2 per reaction. For protein analysis, siRNA protocols were scaled-up proportionally.

### 3D organotypic model of gut mucosa

3D organotypic models were performed in 12-well plates with Transwell® polycarbonate-coated inserts (Corning, UK). Semisolid Collagen gels containing CCD-18Co cells were produced by mixing 70% (v:v) rat tail Collagen-I (2 mg/mL final concentration, 354236, Corning, UK), and 10% (v:v) 10× DMEM (Gibco, UK) on ice. pH was neutralised using 0.5 M NaOH before being mixed with 10% (v:v) FBS and 10% (v:v) CCD-18Co cells (15 000 cells per gel). A total volume of 0.4 mL was pipetted directly onto the inserts and left to polymerise for 1 h at 37°C, before 0.5 mL of a Caco-2 cell suspension (1.2 × 10^5^ cells/mL) were plated on top, and 1.5 mL complete medium was added to the well. Medium was changed the following day and parallel gels were stained with Alexa Fluor anti-F-actin dye (A12379, 1:500, Invitrogen, UK) and Hoechst 33342 (1:500, Invitrogen, UK) and imaged using the IN Cell 2200 microscope (GE Healthcare, UK) to ensure viability and homogenous cell distribution in the gel. Drug treatments were administered on day 3 for 48 h before media were collected for ELISA assays and gel sizes were calculated from light microscopy images taken at 0 and 48 h post-treatment using ImageJ software as a proxy for fibroblast remodelling capacity. Results are from four independent experiments (*n*=4).

### Primary cell isolation

Tissue was collected in DMEM media from strictured small intestine following surgical resection. Resected tissue specimens were washed with Hanks’ Balanced Salt solution (HBSS) supplemented with 0.01% dithiothreitol (DTT) and then HBSS-EDTA (1 mM) for 10 min per wash under agitation at 37°C. For primary myofibroblasts cell cultures, the mucosa was mechanically isolated and only the mucosa was incubated with the collagenase solution. The solution was then filtered through a 100-μM cell strainer and the cell pellet washed twice in PBS. Cells were resuspended in 10 mL DMEM supplemented with 10% heat-inactivated FBS, 1% Penicillin–Streptomycin, 1% L-glutamine, 50 μg/mL gentamycin, and 1 μg/mL amphotericin and transferred to T25 flask. Cells in culture were split once they reached 80% confluency (passage 1) and subcultured once per week thereafter. All experiments were performed using cells between passages 1 and 3. Cell morphology and expression of Collagen-I were used to confirm that cells in culture were fibroblasts. In total, nine human SCD-derived primary CD myofibroblasts cultures were analysed for the present study (*n*=9, Supplementary Table S2). Results in SCD cultures were compared and contrasted with seven NSCD cultures (*n*=7, Supplementary Table S2).

### Immunofluorescence and image analysis

Cells were cultured in 96-well plates (2500 cells per well). Following treatment with ICG-001 and TGF-β1, cells were fixed (4% PFA, 15 min), permeabilised (0.1% Triton-X100, 20 min), and blocked (0.25% BSA, 30 min) prior to incubation overnight at 4°C with the primary antibody; Collagen-I (1:500 dilution; NB600-408, Novus Biologicals) and/or β-catenin (1:100, 610153, BD Bioscience). Cells were subsequently washed (0.1% PBS-Tween) three times for 10 min before addition of a secondary antibody mix comprised of CellMask Deep Red (1:100 000, Applied Biosystems) and DAPI (1 ng/mL), Alexa Fluor® 488-conjugated antirabbit antibody (1:500 dilution; Invitrogen), and/or Alexa Fluor® 555-conjugated antimouse antibody (1:500 dilution; Invitrogen) diluted in blocking reagent for 1.5 h. Cells were washed (0.1% PBS-Tween) three times for 10 min and stored in PBS (200 μL per well) prior to imaging on an IN Cell 2200 Analyser (GE Healthcare). Per well, nine images were taken using a 10× magnification lens. Using the IN Cell Developer software (V1.9), the images were then analysed and density levels of proteins of interest calculated. Mean protein density levels were calculated from all cells (defined using DAPI and cell mask) in all nine images; typically, several hundred cells are analysed per well. Mean density levels were background-corrected by subtracting the values from the secondary antibody-only control.

Immunofluorescence (IF) analysis of CCD-18Co cells treated with 3235-0367 (C1) and Wnt-C59 (C59) was performed independently using a modified protocol. CCD18-Co cells were plated on coverslips at 20 000 cells per well in a 24-well plate. Following treatment, cells were fixed in 4% paraformaldehyde in PBS (Santa Cruz) for 10 min at RT. Permeabilisation was performed using 0.1% Triton X-100 in PBS for 10 min at RT, followed by blocking using 2% BSA, 50 mM glycine, 0.01% NaN3 in PBS for 1 h. Primary antibodies were incubated in blocking buffer overnight at 4°C (Collagen-I, 1:500 dilution; NB600-408, Novus Biologicals or β-catenin, 1:250, 610153, BD Bioscience), followed by incubation with secondary antibodies at 1:500 (AlexaFluor488, AlexaFluor594, Life Technologies). Coverslips were mounted using Vectashield mounting medium with DAPI (Vector Labs). Stained cells were visualised using a Fluorescence microscope (Axioimager D1) with a 20× objective. Intensity analysis was performed using Fiji/ImageJ. For Collagen staining, 8–18 cells per condition and per experiment were selected for intensity measure. For nuclear β-Catenin, 27-120 nuclei were selected for measurement of β-catenin intensity in the nucleus, and for total β-catenin, whole images were thresholded for selection and measurement of the signal in total cell area. Corresponding background intensities were subtracted from all measurements and t-tests were performed on the normalised values for the different experiments (*n*=3).

### Western blotting and ELISAs

Protein was extracted from cells with RIPA buffer (R0278) and protein quantification was determined by DC™ Protein Assay (5000111). Whole cell protein lysates (10–30 μg) were then separated on Bolt 4–12% Bis-Tris plus gels (NW04120BOX) by electrophoresis using the associated manufacturer’s buffers and transferred into polyvinylidene fluoride membrane (IPVH00010). Protein extracts from cells treated with 3235-0367 (C1) and Wnt-C59 (C59) were analysed independently and separated on SDS polyacrylamide gels using a Mini Protean System (BioRad) and transferred to nitrocellulose membranes for 10 min using Trans-Blot Turbo Transfer System (1704271; BioRad). Immunoblotting conditions for each antibody are given below (Supplementary Table S3); the following conditions were optimised for each of the proteins studied: antibody concentration and incubation time, number of washes and time, type of block buffer and concentration, amount of protein, and ECL type. All primary antibodies were incubated overnight at 4°C. Membranes were then washed six times (5 min each) with TBS-T in a shaker (70 rpm). After washing, blots were incubated for 1 h in blocking buffer with HRP-conjugated secondary antibodies diluted and then membranes were developed using chemiluminescence. Image acquisition of the membranes in the chemidoc was performed using the signal accumulation mode. Blots were analysed using computer-assisted scanning densitometry software (Image-Lab 3.0.1, Bio-Rad Laboratories) or Fiji/ImageJ [2]. Pro-Collagen-Iα1 levels measured using a Human Procollagen I alpha 1 DuoSet ELISA as per the manufacturer’s instructions (DY6220-05, R&D Systems).

### TaqMan qPCR and Wnt array

CCD-18Co fibroblasts for the Wnt qPCR array were seeded in 6-well plates overnight in complete media prior to treatment with TGFβ1, as described above. Cells were collected for RNA extraction using the miRNeasy Kit (Qiagen). RNA Samples were reverse transcribed using a Reverse Transcriptase Kit (ABI, USA), cDNA was diluted 1:1 with TaqMan Mastermix and 20 µL added to a 96-well plate containing dried TaqMan probes of 92 Wnt signalling and four endogenous control genes (TaqMan Array Human Wnt Pathway, Cat #4414100, Thermo Scientific, UK). Fold-changes were calculated by the 2⁁-ΔΔCT method. Data were corrected for multiple testing before *P*-values were assigned.

For all other gene expression studies, RNA from CCD-18Co cells were reverse transcribed using the ‘High Capacity RNA-to-cDNA Kit’ (Applied Biosystems). The cDNAs were then diluted 1:10 and incubated with Taqman gene expression probes and Universal Mastermix (Applied Biosystems) on a 7500 System RealTime PCR cycler (ABI). The resultant cycle threshold (Ct) values were normalised to the control genes (e.g. *RPLPO* and/or *ACTB*) using the 2-ΔCt method. Analysis of gene expression in RNA isolated from patient-matched NSCD and SCD (*n*=6, pairs) intestine was performed using RNA isolated from patients as described previously [43].

### TOP/FOP assays

CCD-18Co passage number 34 (p34) were amplified and transduced with a lentivirus vector containing β-catenin-activated reporter (TOP), and its accompanying control reporter system (FOP) provided by Randall Moon [44], reporting Venus fluorescent protein (with hygromycin resistance) at p35, followed by hygromycin selection at 100 µg/mL for 4 days (>95% death in nontransduced control cells). Subsequent experiments were performed through passages p37–p40 in 6-well plates. Cells were plated in 6-well plates and cultured for 48 h in either low serum media (1% FBS) supplemented with TGFβ1 (10 ng/mL), or full serum (10% FBS) CHIR-99021 (CHIR, 5 µM) treatment or their respective vehicle controls (4 mM HCl BSA and DMSO). Cells were then processed for the FACS analysis and we used the FITC filter for VENUS fluorescence protein detection. The FITC mean intensity in different thresholded populations based on % highest FITC (50%, 33%, 15%) for TOP and FOP transduced cells was determined and the TOP/FOP ratio is presented as a fold-change relative to vehicle control from four independent experiments. The differences between treatments and the vehicle control were then determined by a one-sample *t*-test.

### General statistics

Experiments using CCD-18Co cells (qPCR, IF, western blot, and ELISAs) were typically performed in quadruplicate (*n*=4), over four independent cell passages, unless stated otherwise in the figure legend. Differences between treatments were determined by a paired *t*-test to account for different cell passages. In general, data are presented as fold-changes and significant results relative to control are indicated by * symbol (*<0.05, **<0.01, ***<0.001). Solid bars highlight comparisons between specific sets of treatments, and in these instances, * symbol is given above the bar. All qPCR data were log transformed prior to statistical analysis to standardise variance. The effects of treatments in primary myofibroblasts cultures were determined by paired *t*-test to account for difference between cultures.

## Results

### Stricture formation in CD patients is linked to an increase in stromal β-catenin expression and changes in Wnt signalling in myofibroblasts

**Figure 1:**
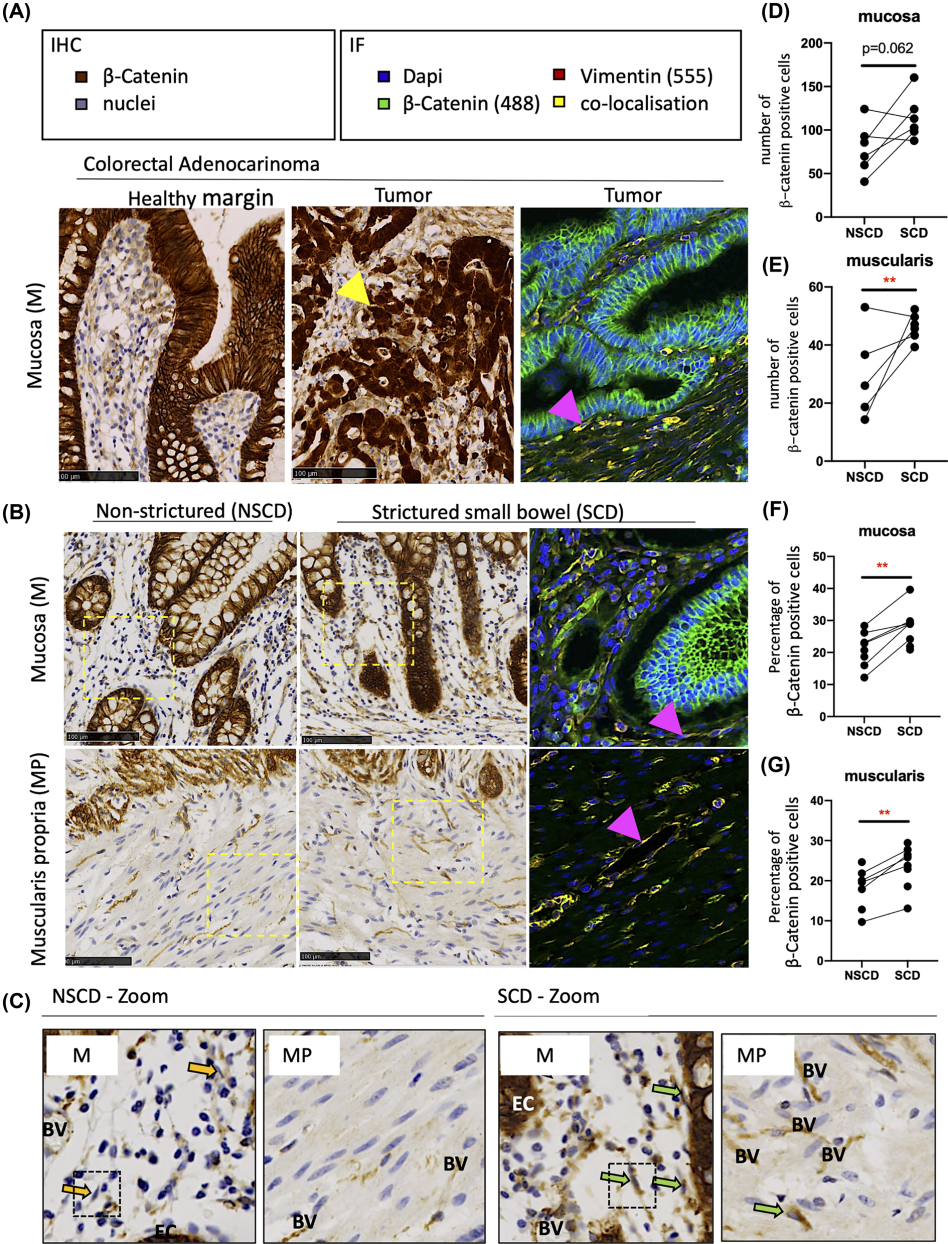
Altered β-catenin expression associated with intestinal fibrosis in SCD patients (**A**) FFPE sections from a colorectal adenocarcinoma block were also used for a positive to control IHC and IF protein analysis, given the link between *APC* mutations in patients, altered Wnt signalling, and changes in β-catenin staining. Nuclear accumulation of β-catenin in epithelial cells (EC) within the tumour are shown by the yellow arrow. The IF further shows clear colocalisation (yellow) of β-catenin (488) and Vimentin (555) in the cytoplasm of fibroblastic cells (pink arrow); DAPI staining demarcates the cell nuclei. (**B**) Representative IHC and IF images of β-catenin in the mucosa (M) overlying SCD and patient-matched NSCD areas (*n*=6) and MP of SCD and NSCD intestine (*n*=5). In the images, positively stained EC, endothelial cells surrounding blood vessels (BV) and stromal cells, including cells that morphologically resembled fibroblasts (indicated by arrows) are observed. (**C**) Enlarged (Zoom) IHC sections from SCD and NSCD mucosa highlighting negatively (yellow arrow) and positively (green arrow) stained fibroblastic cells are highlighted. (**D–G**) Quantification of the total number and percentage of stromal β-catenin-positive cells in the mucosa and the percentage of β-catenin-positive cells in the MP, excluding endothelial cells associated with BV, demonstrates transmural increases in β-catenin throughout SCD tissues. Differences between SCD and NSCD samples were determined by a paired *t*-test. Significant results are indicated by * symbol (*<0.05, **<0.01, ***<0.001).

β-catenin protein levels were analysed by IHC in paired SCD and NSCD FFPE tissue (Figure 1). FFPE tissue from a colorectal adenocarcinoma served as a positive control for β-catenin staining where there was a clear increase in nuclear β-catenin staining relative to the adjacent healthy margin (Figure 1A). SCD and NSCD samples from the same patient were categorised based on histology reports and analysed in a paired fashion. Samples were scored for their extent of fibrosis and ulceration (a marker of inflammation) [42]. SCD samples were characterised by higher fibrosis scores but the extent of ulceration did not differ significantly between SCD and NSCD pairs (Supplementary Table 1).

In the mucosa, strong membranous β-catenin was observed in EC from both SCD and NSCD specimens (Figure 1B). Stromal cells and vimentin-positive cells, including lymphocytes and fibroblasts (arrows), were also positively stained for β-catenin (Figure 1B,C; green arrows). Fibroblasts could be differentiated by their cell morphology, and staining of β-catenin staining was the strongest in SCD-associated fibroblasts relative to their NSCD control (Figure 1C). In the *muscularis*, muscle cells did not express vimentin or β-catenin. However, vimentin-positive fibroblasts expressing β-catenin were observed (arrows). Endothelial cells associated with BV in the mucosa and MP also stained strongly for β-catenin (Figure 1B,C). Scoring of these images confirmed an increase in the number and percentage of stromal β-catenin-positive cells in the mucosa overlying SCD intestine (Figure 1D,F), which mirrored an increase in both the total number and percentage of positive β-catenin cells within the MP of SCD samples (Figure 1E,G).

### Canonical β-catenin activation increases Collagen-I expression in CCD-18Co intestinal fibroblasts in the absence of TGFβ

**Figure 2:**
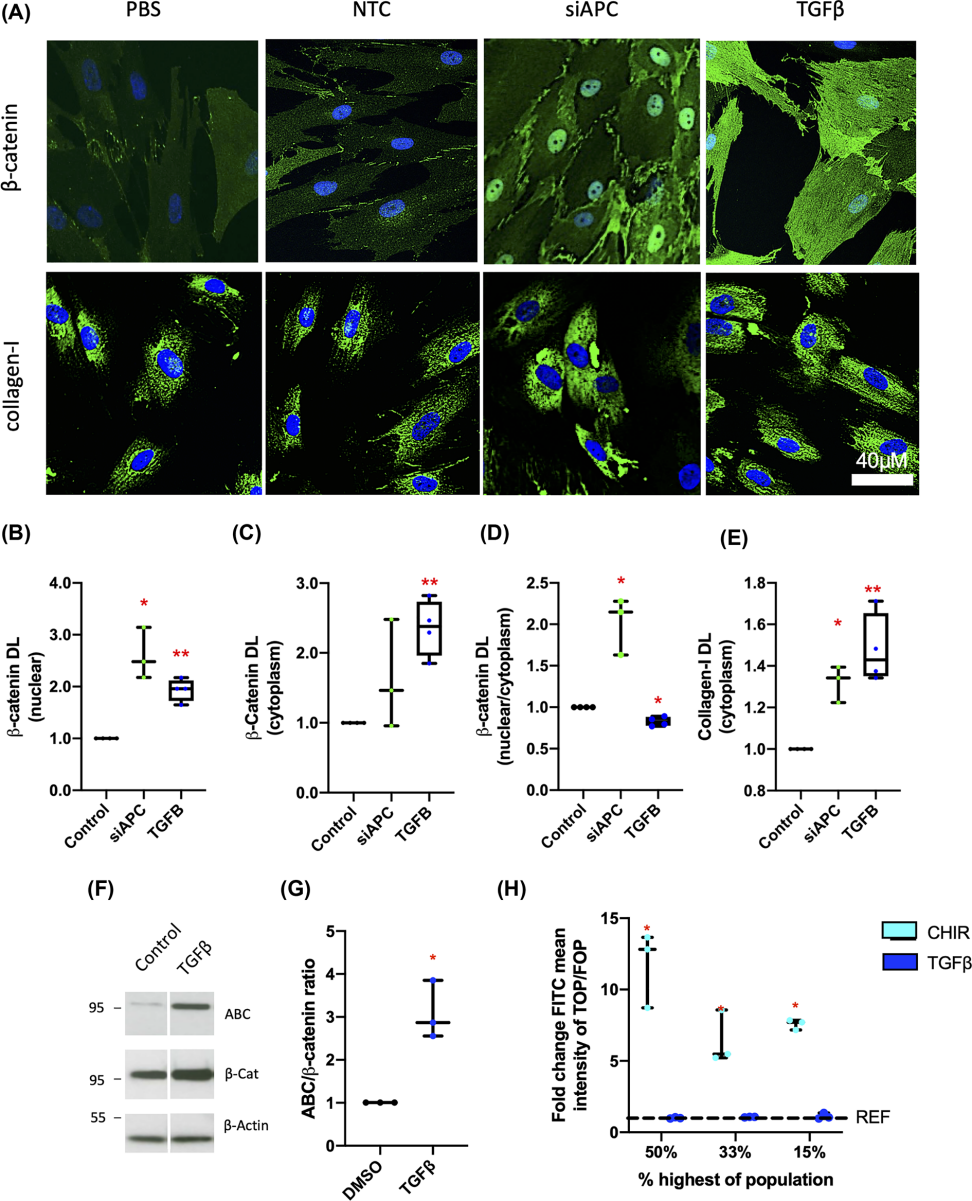
Activation of canonical Wnt β-catenin signalling promotes Collagen-I expression in intestinal fibroblasts in the absence of TGFβ (**A**) Representative IF images of cells stained for DAPI (blue) and β-catenin (green) or Collagen-I (green) 48 h post-transfection with either a negative control siRNA NTC (Cat#1027281) or an *APC*-targeting siRNA (siAPC, Hs_APC_6 Flexitube, Qiagen, UK, *n*=3), or treatment with TGFβ (*n*=4). (**B–D**) The levels of nuclear β-catenin, cytoplasmic β-catenin levels, as well as the cytoplasmic:nuclear ratio of β-catenin were quantified from IF images. (**E**) Cytosolic Collagen-I protein levels were also calculated for all treatment groups. (**F,G**) Representative western blots for activated (dephosphorylated) β-catenin (ABC), total β-catenin, and the loading control β-Actin, along with graphical representation of active:total β-catenin ratio, in CCD-18Co cells treated with TGFβ. (**H**) Results of TOP/FOP assays in CCD-18Co cells treated with TGFβ or the positive control CHIR-99021 (CT99021), a GSK-3 inhibitor, are presented (*n*=3). FITC mean intensity for TOP- and FOP-transduced cells was determined and the TOP/FOP ratio given as a fold-change relative to vehicle control; DL = density levels. Differences between treatments were determined by a paired *t*-test to account for different cell passages. In general, data are presented as fold-changes with panels B–E, G, and H as box plots showing 25th to 75th percentiles, median (horizontal bar), and the smallest and largest value (whiskers). Significant results relative to control are indicated by * symbol (*<0.05, **<0.01, ***<0.001).

To investigate the potential role of Wnt/β-catenin signalling in intestinal fibroblasts, as well as cross-talk with the TGFβ pathway, CCD-18Co human fibroblasts were used as an established model of intestinal fibrosis (e.g. [45–47]). Cells were transfected with a siRNA-targeting APC (siAPC), a negative regulator of β-catenin-dependent Wnt signalling that inhibits the accumulation of β-catenin, or stimulated with TGFβ (Figure 2A). In accord with expectations, analysis of cells by IF revealed that knockdown of *APC* was associated with increased nuclear levels of β-catenin (Figure 2B,C), and an increase in the nuclear:cytoplasmic β-catenin ratio (Figure 2D). Knockdown of *APC* also led to an increase in Collagen-I compared with cells transfected with a nontargeting control (NTC) siRNA (Figure 2E).

TGFβ treatment also increased levels of β-catenin protein (cytoplasm and nucleus) and Collagen-I in intestinal fibroblasts (Figure 2A–E). However, in contrast with cells transfected with siAPC, treatment with TGFβ did not increase the nuclear:cytoplasmic β-catenin ratio (Figure 2D). Instead, TGFβ treatment led to a small reduction in this indicator of canonical Wnt activation (Figure 2D). An increase in cellular β-catenin protein levels was confirmed by western blot; TGFβ also increased the pool of dephosphorylated active β-catenin (Figure 2F,G). In order to study directly Wnt/β-catenin nuclear signalling, we infected CCD-18Co cells with lentiviruses to generate cells expressing a fluorescent protein (Venus) driven by a TCF/LEF-responsive promoter (TOP) or a mutated form of this reporter (FOP) as a control. The FACS analysis of these cells upon stimulation with TGFβ indicated that the observed increase in active β-catenin did not lead to an increase in β-catenin/TCF-dependent transcription (Figure 2H). This was in contrast with treatment with the GSK3 inhibitor CHIR99021 (CHIR), which resulted in a ten-fold increase in the TOP/FOP FITC (Venus) mean intensity. Overall, these data indicate that while TGFβ increases β-catenin levels, this does not lead to activation of β-catenin-dependent transcription in these cells.

### TGFβ up-regulates the expression of noncanonical Wnt mediators FZD8/WNT5B, which are increased in fibrotic strictures in CD patients

**Figure 3:**
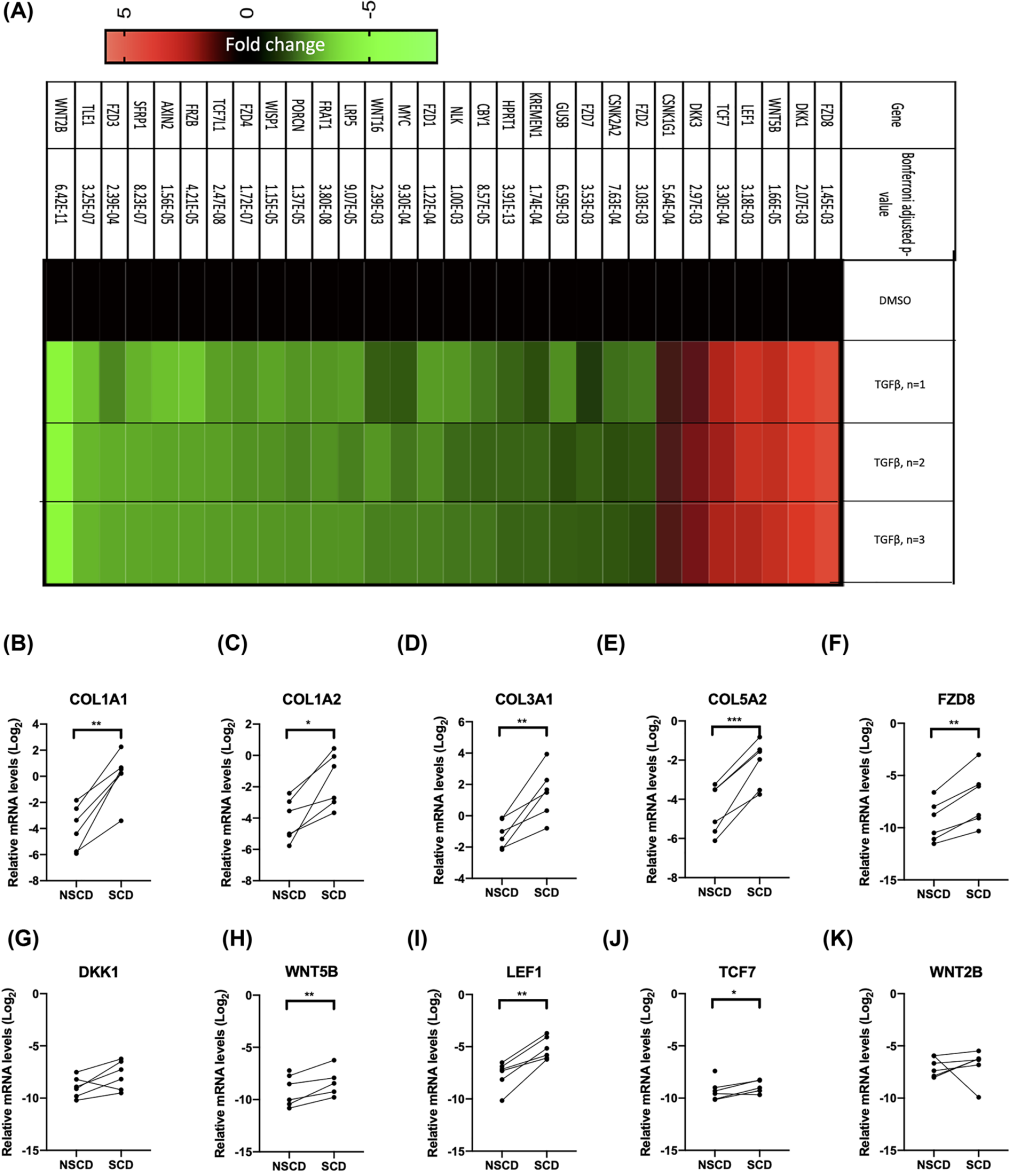
TGFβ increased the expression of FZD8, which is required for TGFβ-mediated up-regulation of Collagen-I (**A**) Genes regulated by TGFβ in the Wnt signalling pathway were identified using a targeted qPCR profiling array (*n*=3) and the data are presented as a heatmap and *P*-values are presented in the figure. The relative fold-change in gene expression in TGFβ-treated cells is given for each of the three replicates. Differences between treatments were determined by *t*-test and corrected for multiple testing. (**B–K**) qPCR quantification of selected collagen and TGFβ-regulated genes in the Wnt-signalling pathway from RNA isolated from the mucosa overlying SCD and patient-matched NSCD intestine (*n*=6). The mRNA levels are normalised to the house-keeping gene RPLPO. Differences between treatments were determined by a paired *t*-test to account for different cell passages. In general, data are presented as fold-changes with panels C–J as box plots showing 25th to 75th percentiles, median (horizontal bar), and the smallest and largest value (whiskers). Significant results relative to control are indicated by * symbol (*<0.05, **<0.01, ***<0.001). A bar indicates specific statistical comparisons.

The data from CCD-18Co cells indicate that activation of Wnt/β-catenin signalling by *APC* gene silencing can promote Collagen-I expression, but that TGFβ treatment does not directly activate this pathway, despite increasing β-catenin protein levels. To investigate whether TGFβ may instead regulate noncanonical Wnt signalling, changes in the expression of Wnt pathway-related genes were investigated using a qPCR gene expression array (Figure 3A). TGFβ did not increase the mRNA levels of canonical Wnt target genes *AXIN2*, which was instead decreased (Figure 3A). *CTNNB1* expression, which encodes β-catenin was unchanged. However, TGFβ treatment did lead to a marked increase in the expression of *FZD8*. The increase in FZD8 was further confirmed at the mRNA and protein level in an independent experiment (Supplementary Figure 1). TGFβ also increased *WNT5B* expression, which encodes a noncanonical ligand that binds to the FZD8 receptor and suppressed expression of the canonical ligand *WNT2B* (Figure 3A). Other genes increased by TGFβ treatment include *LEF1* and *TCF7*, which are part of the β-catenin transcriptional complex (Figure 3A). TGFβ also led to an induction of *DKK1* and *DKK3*, which inhibit canonical Wnt signalling (Figure 3A).

TGFβ-induced changes in Wnt signalling mediators were also found to be pathologically altered in SCD intestine (Figure 3B–K). For this analysis, RNA extracted from paired SCD and NSCD segments of intestine was analysed by qPCR. The five genes with the highest fold-increase in CCD-18Co cells treated with TGFβ (*FZD8*, *DKK1*, *WNT5B*, *LEF1*, and *TCF7*) were selected for analysis; *WNT2B*, which was the most down-regulated gene in TGFβ-treated CCD-18Co fibroblasts was also analysed. Relative to NSCD control tissue, SCD segments of intestine expressed increased levels of *COL1A1*, *COL1A2*, *COL3A1*, and *COL5A2* mRNA (Figure 3B–E). SCD segments of intestine also expressed higher levels of *FZD8*, *WNT5B*, *LEF1*, and *TCF7* mRNA (Figure 3F,H–J) consistent with TGFβ-induced changes observed in CCD-18Co cells. Levels of *DKK1* and *WNT2B* were not significantly different between NSCD and SCD tissue (Figure 3G,K).

### Small-molecule Wnt inhibitors attenuate Collagen-I and inhibit TGFβ-dependent FZD8/Wnt5B signalling in the CCD-18Co cell line

**Figure 4:**
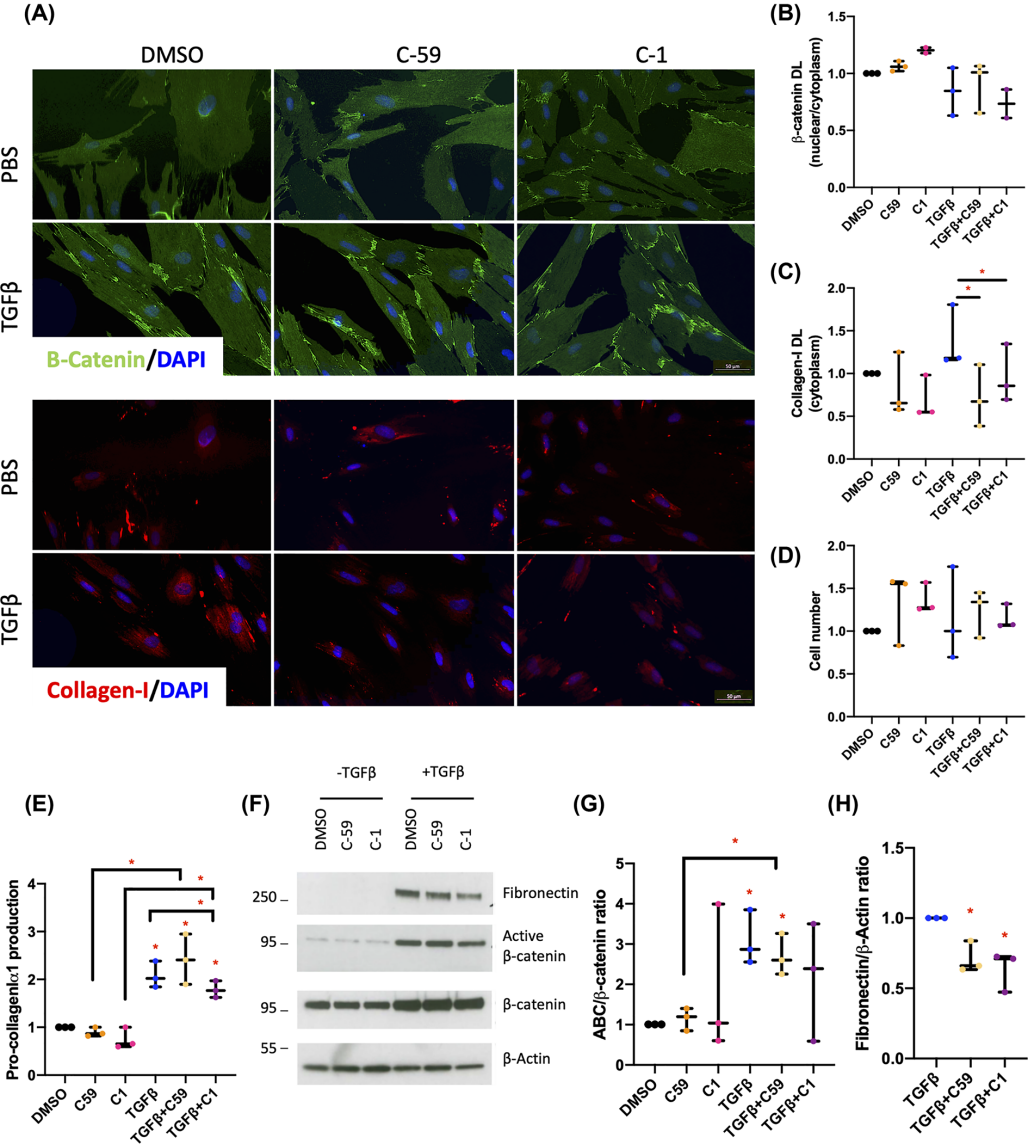
Small-molecule Wnt inhibitors of FZD8 and Wnt5B inhibit Collagen-I expression in intestinal fibroblasts (**A**) β-catenin and Collagen-I protein levels were also determined by IF in treated CCD-18Co cells with Wnt-C59 (C59), 3235-0367 (C1), or the vehicle control (DMSO) in combination with TGFβ. Representative images are provided. (**B**) β-catenin levels are presented as a ratio of the nuclear:cytoplasmic levels of the protein (*n*=2). (**C,D**) Cytosolic Collagen-I levels are also provided (*n*=3), along with cell counts for each treatment (*n*=3). (**E**) Pro-Collagen-Iα1 levels in the media were determined by ELISA (*n*=3). (**F**) Representative western blot showing the effects of 48 h of treatments on Fibronectin, dephosphorylated ABC levels, and total β-catenin protein levels. (**G**) β-catenin protein levels are presented as a ratio of ABC:β-catenin as determined by quantification of the IF images (*n*=3). (**H**) Fibronectin levels are expressed normalised to the loading control (β-Actin); DL = density levels. Differences between treatments were determined by a paired *t*-test to account for different cell passages. In general, data are presented as fold-changes with panels B–E, G, and H as box plots showing 25th to 75th percentiles, median (horizontal bar), and the smallest and largest value (whiskers). Significant results relative to control are indicated by * symbol (*<0.05, **<0.01, ***<0.001). A bar indicates specific statistical comparisons.

The data indicate that both TGFβ-dependent noncanonical FZD8/Wnt5B signalling and TGFβ-independent canonical β-catenin-dependent signalling may play a role in intestinal fibrosis associated with CD. To further investigate the role of these pathways in intestinal fibrosis, we used three small-molecule inhibitors of Wnt signalling: 3235-0367 (C1), a small-molecule inhibitor of FZD8 [26]; Wnt-C59, a porcupine inhibitor that inhibits Wnt ligand secretion and has been shown previously to inhibit ECM protein expression [24,25]; and, ICG-001, which reduces the interaction of β-catenin with the transcriptional co-activator CBP [29]. The dose of C1 used for the present study was experimentally optimised in CCD-18Co cells, using Collagen-I and β-catenin as cell-based readouts of FZD8 activity and a concentration of 10 μM selected (Supplementary Figure S2). At this concentration, inhibition of TGFβ-mediated up-regulation of intracellular Collagen-I was confirmed (Figure 4A,C). Moreover, this reduction correlated with reduced pro-Collagen-Iα1 protein levels in conditioned media (Figure 4E), without significantly affecting cell numbers (Figure 4D) or inhibiting SMAD-driven TGFβ signalling marked by changes in TGFβ1l1 (Supplementary Figure S2). Fibronectin production from cells was also significantly reduced (Figure 4F,H). TGFβ-dependent increases in the active:total β-catenin protein ratio were attenuated by C1 (Figure 4F,G). However, C1 did not have a statistically significant effect on the nuclear:cytoplasmic β-catenin ratio (Figure 4B).

**Figure 5:**
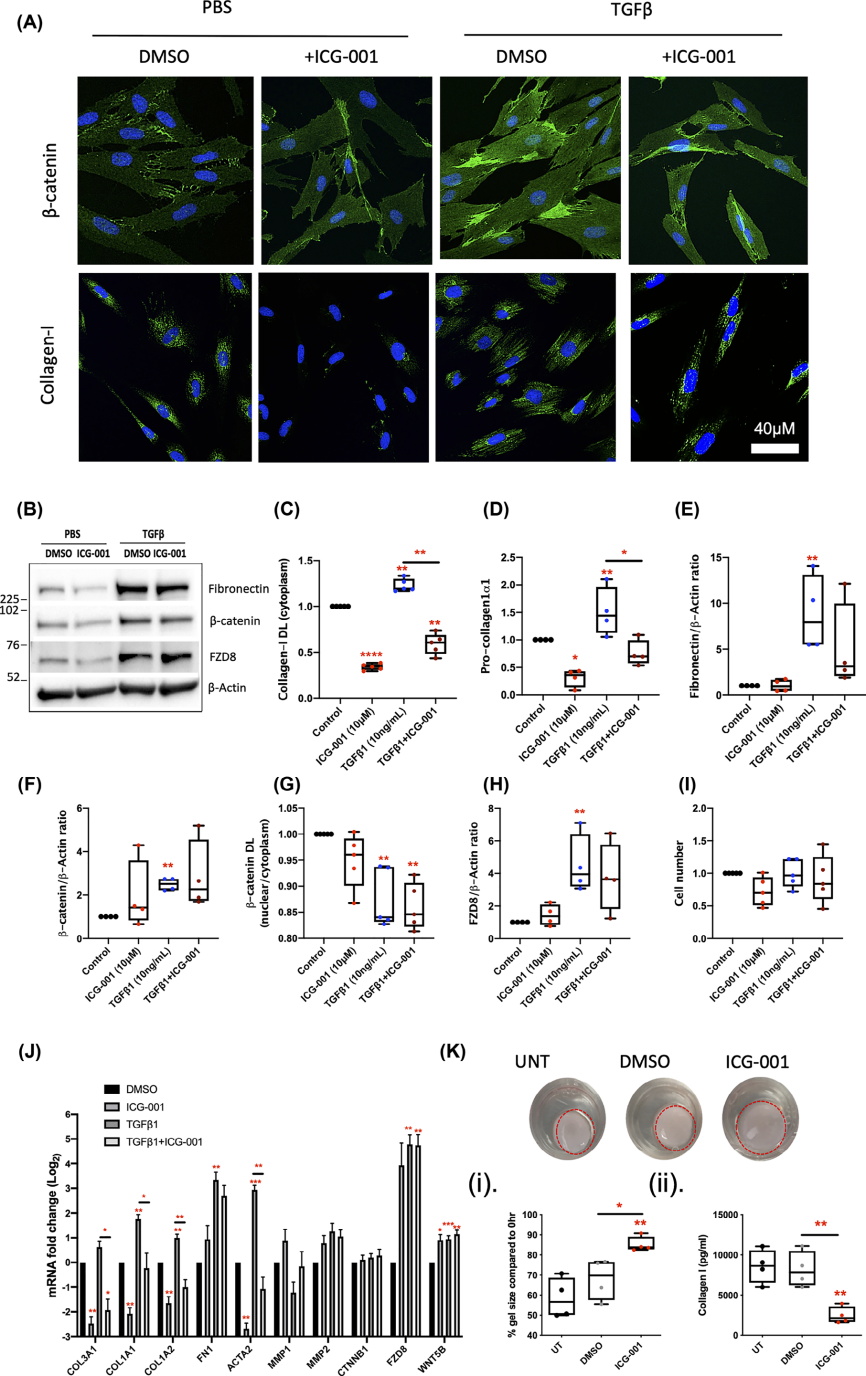
Inhibition of β-catenin-dependent Wnt signalling by ICG-001 blocks both steady state and TGFβ-induced Collagen-I up-regulation (**A**) Levels of β-catenin, Collagen-I, and cell numbers were evaluated by IF (*n*=4). (**B**) Representative western blots for β-catenin, FZD8, and Fibronectin; these data are normalised to the loading control β-Actin (*n*=4). (**C–I**) Protein quantifications from IF and western blots. (**D**) Pro-Collagen-Iα1 levels in the cell media, measured by ELISA, are also provided (*n*=4). (**J**) The ability of ICG-001 to suppress markers of TGFβ-induced myofibroblast activation in CCD-18Co cells was also assessed by qPCR and the data presented in a bar graph (*n*=4). (**K**) ICG-001 effects on gel remodelling and pro-Collagen-Iα1 were also confirmed in a 3D organotypic model in the absence of TGFβ (*n*=4); DL = density levels. Differences between treatments were determined by a paired *t*-test to account for different cell passages. In general, data are presented as fold-changes with panels C–I and K(i–ii) as box plots showing 25th to 75th percentiles, median (horizontal bar), and the smallest and largest value (whiskers). Panel J shows mean ± SEM. Significant results relative to control are indicated by * symbol (*<0.05, **<0.01, ***<0.001). A bar indicates specific statistical comparisons.

Blocking Wnt ligand production by treatment with C59 had similar effects to treatment with C1 on intracellular Collagen-I protein levels and fibronectin (Figure 4A,C,F,H). Although, in contrast with C1, changes in intracellular Collagen-I were not reflected by a change in pro-Collagen-Iα1 protein levels in conditioned media (Figure 4E). C59 treatment had no effect on TGFβ-dependent changes in the active:total β-catenin protein ratio or the nuclear:cytoplasmic β-catenin ratio in treated cells (Figure 5B,F,G). Importantly, the effects of C1 treatment were replicated by targeted siRNA-mediated knockdown of FZD8 in TGFβ-stimulated cells (Supplementary Figure S1).

### Inhibition of TGFβ-independent β-catenin-driven canonical Wnt signalling

The effects of C1 were also compared with ICG-001 (10 μM), which inhibits canonical Wnt signalling by disrupting the interaction of β-catenin with CBP [29]. Like C1, the dose of ICG-001 was experimentally optimised to inhibit Collagen-I protein levels in the absence of detrimental effects on cell number (Supplementary Figure S2). IF and qPCR analysis of CCD-18Co cells treated with ICG-001 identified a reduction in Collagen-I protein (Figure 5A,C,J), and an ELISA confirmed similar changes in pro-Collagen-1α1 production from cells (Figure 5D). At the mRNA levels, ICG-001 also reduced *COL3A1* and *ACTA2* expression and limited their up-regulation by TGFβ in CCD-18Co fibroblasts (Figure 5J). In comparison with C1 and C59 (Figure 4), ICG-001 inhibited TGFβ-induced up-regulation of Collagen-I to a greater extent. However, ICG-001 also inhibited steady-state Collagen-I expression (Figure 5A,C,J) consistent with the hypothesis that this pathway is TGFβ-independent. Like C1, ICG-001 had no effect on cell number (Figure 5I). The ICG-001-dependent reduction in pro-Collagen-1α1 production in the absence of TGFβ was further confirmed in 3D assays, where ICG-001 also inhibited contraction, a marker of fibroblast activity (Figure 5K(i–ii)). The mRNA levels of *MMP1*, an interstitial collagenase, and *FN1*, which encodes fibronectin, remained unchanged (Figure 5J). Fibronectin protein levels were not statistically affected either, despite mean levels of fibronectin being lower in TGFβ-stimulated cells treated with ICG-001 (Figure 5B,E).

ICG-001 did not affect β-catenin protein levels, the nuclear:cytoplasmic β-catenin ratio or CTNNB1 mRNA expression (Figure 5A,B,F,G,J), consistent with its function as an inhibitor of β-catenin-dependent transcription, rather than a regulator of β-catenin expression. Moreover, ICG-001 increased mean levels of FZD8 (mRNA and protein), although this was not significant (Figure 5B,H,J). ICG-001 also increased WNT5B mRNA expression (Figure 5J), suggesting ICG-001 effects are independent of TGFβ-induced changes in the WNT5B/FZD8 axis. Taken together, the data highlight the potential to inhibit TGFβ-dependent regulation of ECM using small-molecule inhibitors of either canonical or noncanonical Wnt signalling.

### Validation of antifibrotic Wnt inhibitors in intestinal myofibroblast cultures isolated from surgically resected fibrotic strictures

Of the three inhibitors tested, C1 and ICG-001 were selected for further investigation in primary intestinal myofibroblast cultures isolated directly from surgically resected SCD tissue (Supplementary Table S2). Based on the studies of CCD-18Co cells and the pathologically important increase in Collagen-I deposition in strictured intestine, the primary endpoints for C1 and ICG-001 in primary CD cells were changes in Collagen-I protein levels determined by IF and ELISA. The impact of both drugs on β-catenin levels and cell number were also determined.

**Figure 6:**
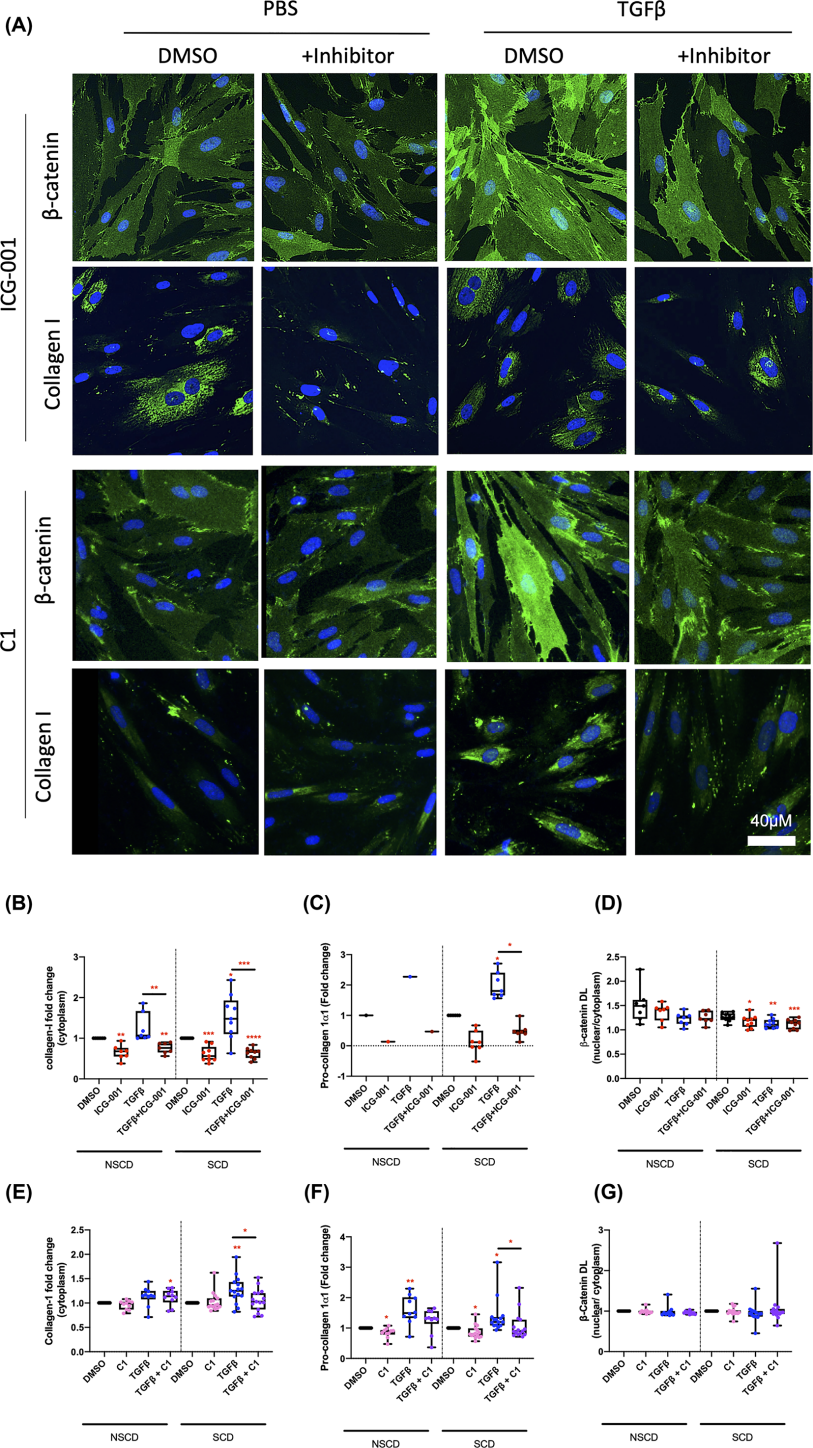
ICG-001, β-catenin/CBP inhibitor reduces Collagen-I expression in primary CD myofibroblast cultures (**A**) Representative IF from primary CD cultures (*n*=7 and *n*=9 NSCD and SCD cultures, respectively) treated with and without TGFβ in combination with ICG-001 and C1. (**B–G**) Protein quantifications from IF images and ELISA (conditioned media) from ICG-001 and C1 treated myofibroblast cultures. *DL = density levels. Differences between treatments were determined by a paired *t*-test to account for different cell passages. Differences between SCD and NSCD cultures determined by an unpaired *t*-test assuming equal variance. Significant results relative to control are indicated by * symbol (*<0.05, **<0.01, ***<0.001). A bar indicates specific statistical comparisons.

For assessment of ICG-001, 16 cultures were screened from seven CD patients; seven cultures were established from NSCD tissue and nine cultures were established from SCD tissue. In some instances, multiple cultures were derived from a patient from phenotypically distinct segments of intestine, e.g. SCD and NSCD areas. Each culture was treated as an independent replicate. Protein analysis (IF and ELISA) confirmed the ability of ICG-001 to suppress baseline and TGFβ-induced Collagen-I expression within the cells and production of pro-Collagen Iα1 from cells (Figure 6A–C). IF also indicated ICG-001 suppressed these markers in both NSCD and SCD lines to a similar extent (Figure 6B,C). In line with its action as an inhibitor of canonical Wnt signalling, ICG-001 also lowered the nuclear:cytoplasmic β-catenin ratio within the cells (Figure 6D). Extended phenotyping of ICG-001-treated cells (mRNA and western blot) also identified changes in other profibrotic mediators, e.g. fibronectin (Supplementary Figure S3).

**Figure 7:**
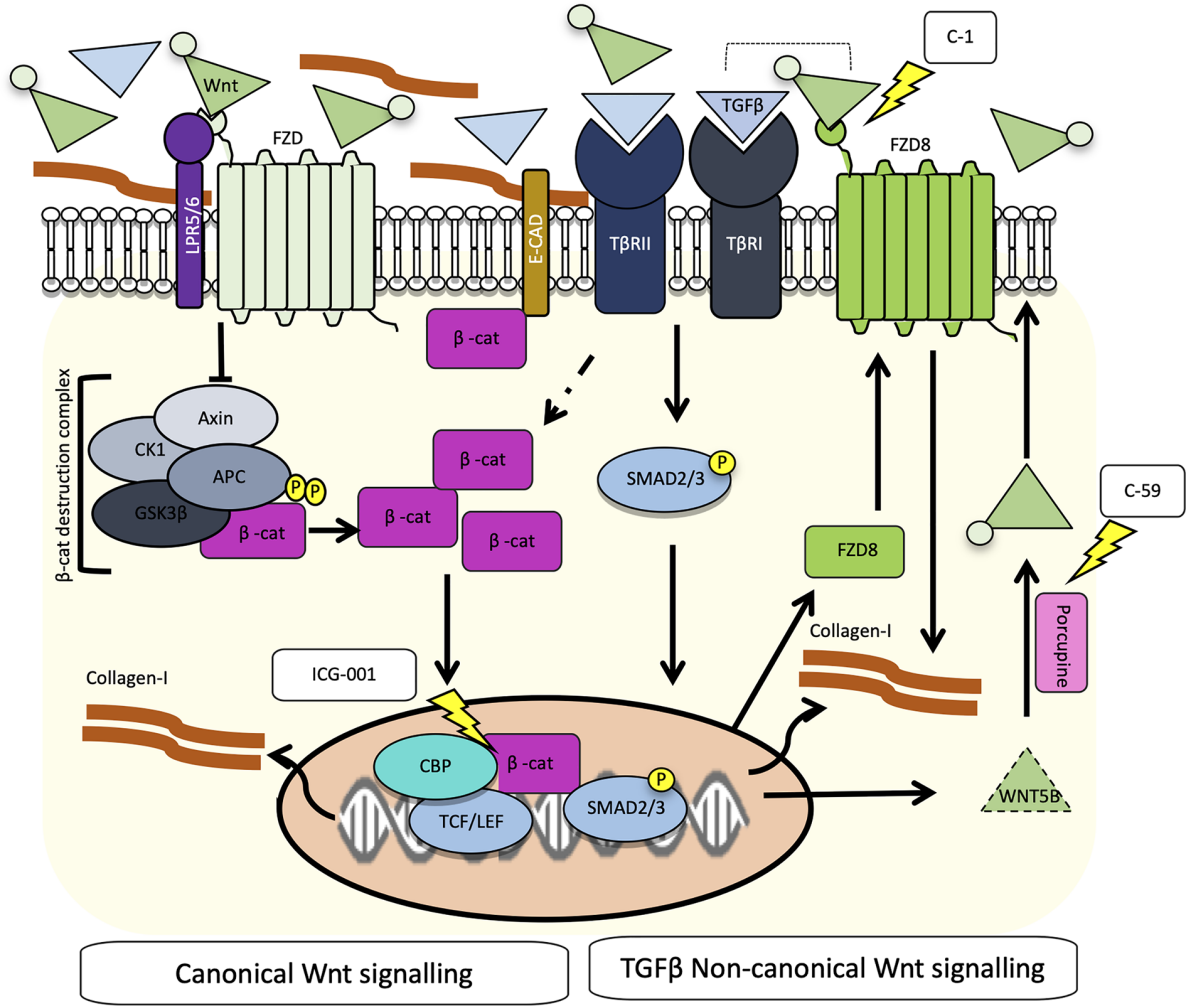
Model for Wnt-mediated fibrosis in CD Binding of Wnt ligands to FZD receptors leads to canonical activation, inhibition of the β-catenin destruction complex, and an increase in dephosphorylated active β-catenin. β-catenin is then free to translocate to the nucleus, where along with cofactors such as CBP, it activates TCF/LEF-dependent gene transcription and as shown in the present study up-regulates Collagen-I. Conversely, inhibitors of the β-catenin-dependent transcription such as ICG-001 reduce Collagen-I expression. ICG-001 can also disrupt interactions between Smad3 and β-catenin are CBP-dependent [34]. In intestinal fibroblasts, the profibrotic cytokine TGFβ does not directly activate the canonical Wnt pathway in intestinal fibroblasts. Instead, TGFβ promotes noncanonical Wnt signalling mediated by FZD8/Wnt5B. Inhibiting either the FZD8 receptor with a small-molecule inhibitor (C1; 3235-0367) or blocking Wnt ligand production in TGFβ-stimulated fibroblasts, which also results in reduced Collagen-I expression from intestinal fibroblasts. These two parallel pathways can regulate Collagen-I independently but there is also potential for cross-talk, given that the TGFβR complex can associate with the FZD8 receptor and TGFβ1 can promote the accumulation of β-catenin in fibroblasts, which may prime cells to be more Wnt-responsive.

For testing of C1, primary cells from five CD cell cultures: two cultures from NSCD tissue and three cultures from SCD tissue were used. For each culture, experiments were repeated five times over independent passages. The results demonstrate that unlike ICG-001, C1 selectively blocks TGFβ-induced Collagen-I expression and production of pro-Collagen-Iα1 (Figure 6A,E,F) but does not alter the nuclear:cytoplasmic β-catenin ratio within the cells (Figure 6G). The results for C1 treatment in primary cells are consistent with the data from CCD-18Co cells and support our hypothesis that while canonical Wnt signalling is independent of TGFβ, noncanonical FZD8-mediated Wnt signalling is required for TGFβ-Wnt cross-talk in intestinal fibroblasts. A model for Wnt-mediated fibrosis and the use of small-molecule Wnt inhibitors in CD is shown in Figure 7.

## Discussion

In the present study, we have demonstrated increased β-catenin expression in SCD tissue by IHC. Wnt signalling pathway components, including FZD8 and WNT5B, were also up-regulated in SCD tissue. Furthermore, we identify small-molecule inhibitors of FZD8 (3235-0367 (C1)), Porcupine (Wnt-C59), and β-catenin (ICG-001) as potential novel therapies for intestinal fibrosis in CD.

Our data are supported by the previous reports of increased nuclear β-catenin staining in FFPE CD fibrotic lesions relative to healthy controls [40]. However, our paired study design ensures that potential confounding variables such as genetics, medications, or disease duration could not account for the observed increases in β-catenin-positive cells in SCD tissue. Moreover, pathological assessment of the tissue also indicated that SCD and NSCD samples were relatively well matched for the extent of ulceration, a marker of inflammation. They are also consistent with data from fibrotic diseases of other organs [15,19].

A direct link between β-catenin-dependent Wnt signalling and Collagen-I was demonstrated *in vitro* using the CCD-18Co human intestinal fibroblast cell line, an established model of intestinal fibrosis [e.g. 45–47]. Here, we showed that increasing the nuclear:cytoplasmic ratio of β-catenin by silencing the *APC* gene was sufficient to induce Collagen-I production. Moreover, treatment with ICG-001, which inhibits β-catenin/CBP-dependent transcription [29], reduced steady-state Collagen-I expression, and prevented Collagen-I up-regulation by TGFβ. Importantly, these results were validated in primary CD myofibroblast cultures derived from surgically resected CD specimens.

Our data are consistent with the previous reports demonstrating ICG-001 inhibits fibrosis in liver [32,48], kidney [49], and lung [30,50]. Encouragingly, mouse models and clinical trials further indicate that ICG-001 is a relatively safe drug. In models of colorectal cancer, ICG-001 induces apoptosis in colon cancer cells, but not in normal colon cells [22]. ICG-001 also reduced polyp formation in the Min mouse, which has a germline mutation in the *APC* gene, with no overt toxicity [29]. These findings are important as they suggest that ICG-001 may not disrupt normal intestinal EC function, a key concern with Wnt inhibitors, given the importance of Wnt signalling in EC homeostasis.

Moreover, in models of kidney fibrosis, blocking β-catenin/TCF-dependent signalling with ICG-001 not only prevented fibrosis but ICG-001 redirected β-catenin and promoted β-catenin/FOXO1 signalling and thus inhibited inflammation, suggesting ICG-001 may offer a dual benefit for CD patients [51]. A second-generation β-catenin/CBP inhibitor, PRI-724 reduced hepatic collagen deposition, improved histological fibrosis, and was well tolerated in a phase I trial in HCV cirrhosis [33]. ICG-001 and PRI-724 have not been tested in mouse models of IBD. However, the absence of suitable murine models of stricturing CD may limit this approach as a means of assessing efficacy of these drugs *in vivo*. While *in vitro* models of intestinal fibrosis cannot model the complex intestinal architecture, *in vivo* rodent models do not fully recapitulate human disease and have given little insight into mechanisms with translational potential [8]. Development of physiologically relevant human *in vitro* models represents a realistic alternative route to identifying new therapeutic drugs.

In the present study, we also describe cross-talk between the TGFβ and Wnt signalling pathways. TGFβ has previously been reported to induce fibrosis both via regulating β‐catenin‐dependent [52], and -independent Wnt signalling [23]. Thus, the interaction between the TGFβ and Wnt signalling pathways is likely to be cell-type and context-dependent. In CCD-18Co cells, TGFβ post-transcriptionally increased both total and ‘active’ β-catenin protein levels in a Wnt ligand-independent manner. However, TGFβ did not increase the nuclear:cytoplasmic ratio of β-catenin, increase TCF/LEF transcription in lentiviral-transduced cells reporting TOP/FOP transcriptional activity, or increase the expression of the β-catenin/Tcf target gene *AXIN2* in intestinal fibroblasts. This suggests that the TGFβ-induced increases in β-catenin are not sufficient to activate canonical Wnt signalling. Instead, TGFβ treatment markedly increased the expression of components of noncanonical Wnt signalling pathways, e.g. FZD8 and its ligand WNT5B, which were both up-regulated in SCD tissue relative to paired NSCD tissue. As far as we are aware, this is the first report of altered FZD8/WNT5B expression in SCD tissue and provides important patient-based validation of a role for FZD8/WNT5B in SCD. Although we cannot directly infer that these changes are TGFβ-driven, this hypothesis is supported by the mechanistic *in vitro* data presented here and the wide-spread reports of increased TGFβ in SCD tissue. Notably, TGFβ has also been shown to up-regulate FZD8 in mesenchymal cells derived from human intestinal organoids [53].

The FZD8 receptor has been shown previously to associate with the TGFβ receptor complex, highlighting it as potential mediator of cross-talk between the TGFβ and Wnt signalling pathways [54]. The observed up-regulation is also consistent with the previous studies linking high FZD8 levels and WNT5B to activation of noncanonical Wnt signalling during lung and liver fibrosis [21]. In CCD-18Co cells, FZD8 directly contributed to TGFβ-mediated stabilisation of β-catenin protein and up-regulation of Collagen-I, although the precise mechanism is unclear. However, given TGFβ does not activate β-catenin/Tcf-dependent transcription in these cells, activation of a noncanonical Wnt pathway seems more likely.

Inhibitors of the WNT and FZD8 signalling were also shown to limit Collagen-I expression in CCD-18Co cells and CD-derived myofibroblasts, highlighting the therapeutic potential of targeting this signalling axis. FZD8 is a particularly attractive therapeutic target given it selectively targets TGFβ-induced Collagen-I and does not influence basal expression, highlighting potential selectivity for pathological activated myofibroblasts. This is likely associated with its low basal expression in intestinal fibroblasts and its marked induction in TGFβ-activated cells. Moreover, targeting FZD8 at the concentration used here (10 μM) does not appear to disrupt canonical SMAD-driven TGFβ-signalling, which, given its important role in suppressing inflammation in CD, is a positive finding. The dose of FZD8 inhibitor used is also consistent with other studies using cell and NMR-based methods that demonstrate micromolar effects for C1 (IC_50_ 7 μM, *K*_d_ 2.5 μM) [26].

The FZD8 inhibitor C1 used in the present study blocks FZD8-dependent Wnt signalling by binding to the conserved cysteine-rich domain of FZD8 [26,27]. This drug has previously been shown to reduce migration of cancer cells [54], and ECM production (Collagen-1 and Fibronectin) by trabecular meshwork cells [27]. Here, we have shown that the FZD8 inhibitor reduces TGFβ induction of fibronectin, Collagen-I and, to some extent, β-catenin in CCD-18Co intestinal fibroblasts. The results with C1 were also supported by siRNA mediated knockdown studies of *FZD8* in CCD-18Co fibroblasts reported here, and studies showing that FZD8‐deficient mice are resistant to bleomycin‐induced lung fibrosis [23].

C59, a porcupine inhibitor that blocks Wnt ligand production, was used to target Wnt5B expression in TGFβ-treated fibroblasts. In accord with the previous reports, C59 inhibited ECM gene expression, but less potently than C1. These effects on ECM protein levels were independent of changes in β‐catenin and support a noncanonical action. In this context, this would be consistent with the antifibrotic effects of blocking WNT5B/FZD8 signalling. However, as Wnt-C59 inhibits secretion of all Wnt family members, we cannot directly link its actions to a specific Wnt ligand. Therefore, targeting the FZD8 receptor selectively may offer greater specificity. To our knowledge the FZD8 inhibitor, C1 has not yet been tested as part of a clinical trial. This will be important in determining its potential as a therapy, given the safety concerns surrounding bone fragility that led to termination of a dose-escalation phase 1b trial of Vantictumab, a monoclonal antibody that binds FZD1, FZD2, FZD5, FZD7, and FZD8, in metastatic pancreatic cancer patients [55–57].

In summary, our data highlight complex interactions between canonical Wnt and TGFβ-mediated noncanonical FZD8-Wnt5B-mediated signalling pathways in the context of intestinal fibrosis in CD. Both can induce fibroblasts to increase production of ECM proteins, e.g. Collagen-I, and small-molecule inhibitors of both can attenuate this response, and therefore offer opportunities for therapeutic intervention. TGFβ promotes noncanonical Wnt signalling, but does not directly induce canonical Wnt signalling in intestinal fibroblasts despite increasing the protein levels of β‐catenin. However, it is possible that the higher levels of β‐catenin protein observed in TGFβ-stimulated fibroblasts primes those cells to be more Wnt responsive *in vivo*, where Wnt ligands in the local microenvironment can initiate β‐catenin-dependent canonical Wnt signalling.

A proposed model for Wnt-mediated fibrosis in CD is presented (Figure 7). A similar model of TGFβ-induced Wnt activation has been proposed in models of lung fibrosis where both canonical and noncanonical changes in Wnt signalling have been implicated [23]. Moreover, it is consistent with studies in systemic sclerosis (SSc) where SSc fibroblasts do not display autonomous increases in Wnt activation in culture but are primed by TGFβ to be hyper-responsive to Wnt ligands [58]. In the SSc fibroblasts, this mechanism involves the down-regulation of Axin2 [58]. Consistent with the study of Gillespie et al. [57], the results from the Wnt qPCR gene expression array in CCD-18Co fibroblasts showed that TGFβ reduced expression of *AXIN2*, a Wnt-inducible component of the β‐catenin destruction complex. This mechanism could contribute to the stabilisation of β‐catenin in TGFβ-treated intestinal fibroblasts.

## Clinical perspectives

- Currently, there are no specific antifibrotic therapies for stricturing disease in CD; therefore, surgery remains the cornerstone of treatment despite its costs, risks, and the subsequent problems associated with disease recurrence.
- Hence, there is an urgent need for new therapies to transform the way patients with strictures are treated and to improve their quality of life, based on a better understanding of the underlying molecular mechanism. Our data highlight small-molecule Wnt inhibitors, in particular ICG-001 and C1, for further investigation as potential antifibrotic drugs to treat intestinal fibrosis in CD.

## Supplementary Material

Supplementary Figures S1-S3 and Tables S1-S3

## Data Availability

The data underlying the present article will be shared on request to the corresponding authors.

## Abbreviations

ABC: activated β-catenin
BV: blood vessel
CBP: CREB-binding protein
CD: Crohns disease
COL1A1: collagen type I alpha 1 chain
DAPI: 4′,6-diamidino-2-phenylindole
DL: density level
EC: epithelial cell
ECM: extracellular matrix
FBS: fetal bovine serum
FFPE: formalin-fixed paraffin-embedded
FITC: fluorescein isothiocyanate
FZD: Frizzled
IBD: inflammatory bowel disease
IF: immunofluorescent
IHC: immunohistochemistry
MMP: matrix metalloproteinases
MP: muscularis propria
NSCD: nonstricturing CD
NTC: nontargeting control
PFA: paraformaldehyde
SCD: stricturing CD
SEM: standard error of mean
siAPC: siRNA targeting APC
SSc: systemic sclerosis
TCF/LEF: T-cell factor/lymphoid enhancer factor
TGFβ: transforming growth factor beta 1
Wnt: wingless-Int-1
