# Supplementary material for: Small-molecule Wnt inhibitors are a potential novel therapy for intestinal fibrosis in Crohns disease
Source: Clin Sci (Lond). 2022 Oct 14;136(19):1405–23. doi: 10.1042/CS20210889 (PMC9583737; doi:10.1042/CS20210889)
Supplement: Supplementary Figures S1-S3 and Tables S1-S3 [file CS-2021-0889_supp.pdf]

Supplementary Figures

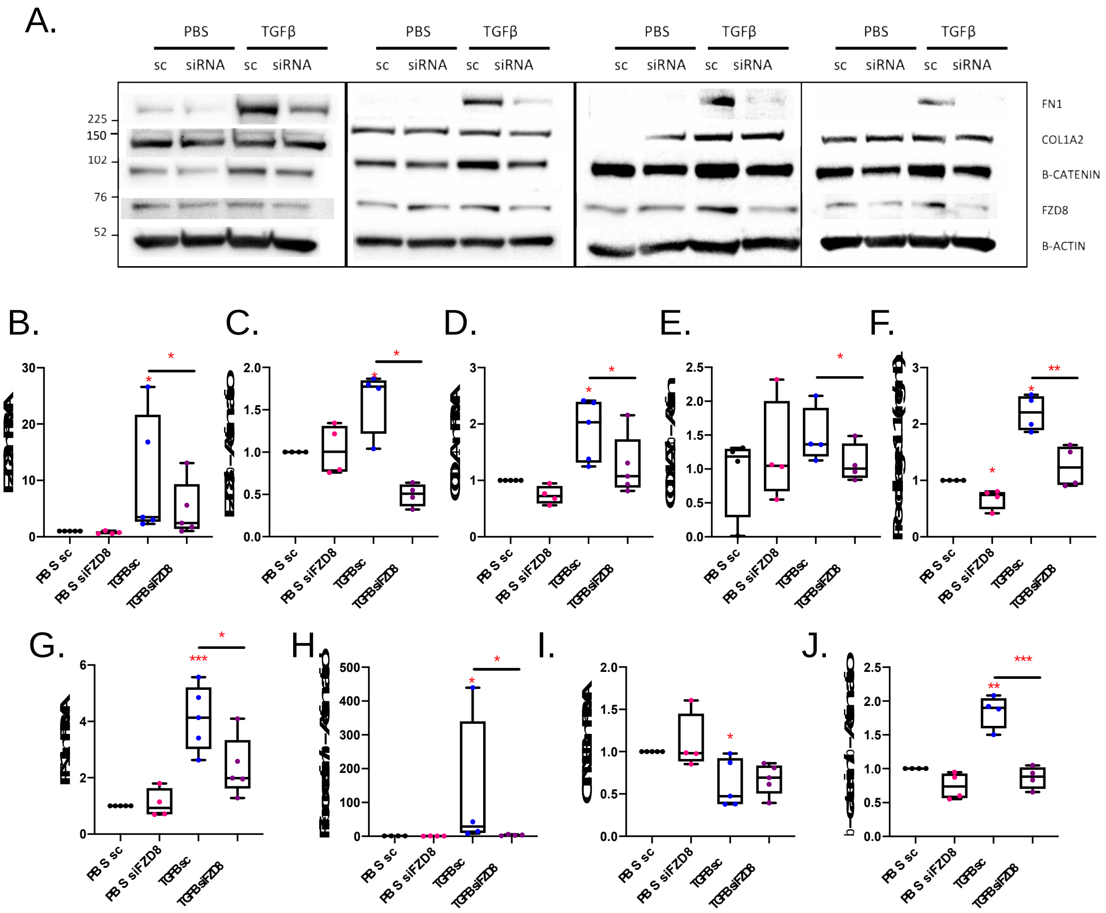

Supplementary Figure S1. A) Representative western blots from CCD-18Co cells transiently transfected with a scrambled (sc) non-targeting control siRNA or siRNA targeting FZD8 (siFZD8). (B-C) Successful FZD8 knockdown was confirmed by qPCR and western blot in TGFβ treated cells (n=4). (D-E) FZD8 knockdown blocked TGFβ mediated up-regulation of COL1A1 mRNA and inhibited production of Pro-collagen-Iα1 protein in conditioned cell media (n=4, respectively). (F-G) FZD8 knockdown also impaired TGFβ's ability to up-regulate Fibronectin mRNA and protein (n=4, respectively). (H-I) FZD8 deficiency did not affect β-catenin (CTNNB1) mRNA levels, but did inhibit TGFβ-dependent stabilisation on β-catenin protein levels (n=4).

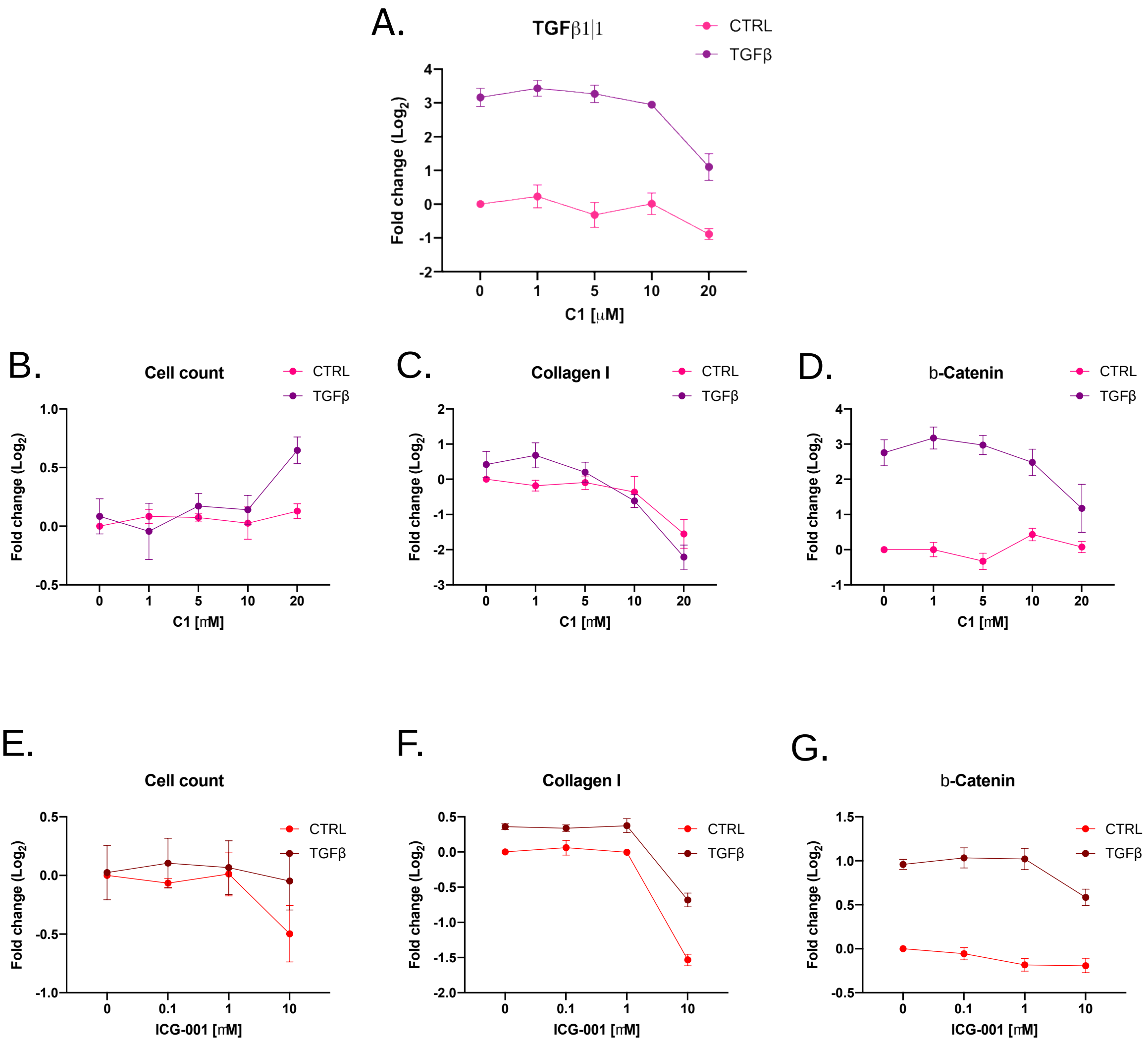

Supplementary Figure S2. (A) TGFβ treatment induces expression of the SMAD target TGFβ1|1. (B-D) Quantification of cell number, Collagen-I and β-catenin protein levels from IF images of CCD-18Co cells treated with C1 or (E-G) ICG-001.

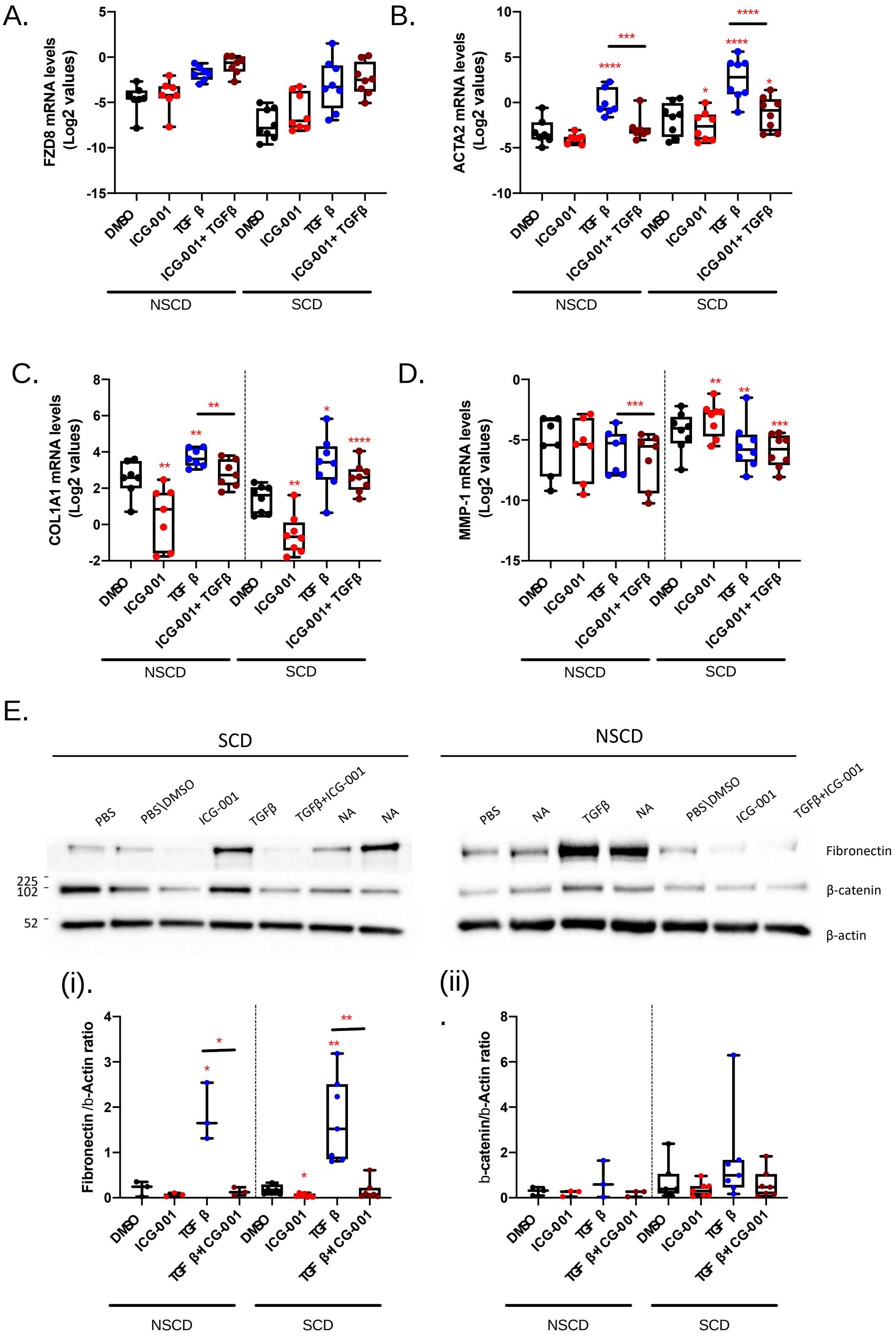

Supplementary Figure S3. (A-D) analysis of mRNA levels of selected genes from primary CD myofibroblasts derived from NSCD and SCD intestine. (E) western blot analysis of fibronectin and β-catenin with associated quantification of protein levels from primary CD myofibroblasts derived from NSCD and SCD intestine**.**

Supplementary Tables

Supplementary Table S1. Stricturing CD FFPE cohort characteristics

Pathology ID	AGE	Gender	Age at diagnosis	Montreal	CRP	Active disease at sample collection	Medications	Block	Fibrosis score	ulceration score
16S005439	26	F	15	A1L1B3	52	yes	thiopurine	NSCD	7	0
								SCD	11	0
16S008009	35	F	23	A2L1B2	<5	yes	adalimumab, methotrexate	NSCD	9	0
								SCD	11	5
10S014520	x	M	16	A1L1L4B2		yes	x	NSCD	6	0
								SCD	8	0
10s40494	27	F	20	A2L1B2	<5	no	none	NSCD	6	0
								SCD	10	0
16S042693	71	F	63	A3L1L4B2	73	yes	adalimumab	NSCD	0	0
								SCD	10	6
17S013903	x	x	x	x	x	x	x	NSCD	5	0
								SCD	12	6
p value NSCD Vs SCD									0.0312	0.125

*All blocks were reviewed by a pathologist who scored the extent of ulceration and fibrosis. Difference between Non-strictured CD blocks (NSCD) and strictured CD blocks (SCD) were determined using a Wilcoxon test. CRP= C-reactive protein. X = data not available

Supplementary Table S2. Primary CD myofibroblasts cohort characteristics

Sample ID	NSCD cultures (n=7)	SCD cultures (n=9)	AGE	Gender	Age at diagnosis	Montreal	CRP	Active disease	Medications
19RLH017	0	2	25	M	11	A1L3L4B1p	20	yes	thiopurine
19RLH016	0	3	28	M	12	A1L3B2P	37	yes	none
19RLH0019	0	2	35	M	26	A2L1L4B3	18	yes	adalimumab
19RLH023	2	1	27	M	12	A1L3B2p	13	yes	vedolizumab
19RLH024	3	0	24	F	18	A2L2L4B1	58	yes	none
19RLH026	0	1	26	F	16	A1L3B3p	61	yes	adalimumab, thiopurine
19RLH031	2	0	33	M	16	A1L3B1p	2	No	adalimumab, thiopurine

*No patients were taking steroids at the time of collection. From some patients' multiple independent strictures were harvested and cell cultures derived.

Supplementary Table S3. Source of antibody and immuno-blotting conditions. Dako secondary antibodies: Goat-α-Rabbit Immunoglobulins/HRP (DAKO, P0448), Rabbit-α-Mouse Immunoglobulins/HRP (DAKO, P0260), or the appropriate HRP-conjugated secondary antibodies from Jackson ImmunoResearch. ECL reagent: Supersignal West Dura (Thermo Scientific, 34075), Pierce ECL Western (Thermo Scientific, 32106) or Amersham ECL Western Blotting Detection Reagents, GE Healthcare

Target	Company	Species	Blocking buffer	Primary Ab dilution	Secondary Ab dilution	ECL
β-catenin	BD Transduction, 610154	Mouse	5% BSA	1/1000	1/2000, Dako	Thermo, 32106
Active β-catenin	Cell Signalling, 8814	Rabbit	5% milk	1/1000	1/2000, Dako	Thermo, 32106
Active β-catenin	Millipore,05-665-25UG	Mouse	3% BSA	1/1000	1/20000, Jackson	Amersham
Fibronectin	AbCam, ab154210	Mouse	5% BSA	1/500	1/1000, Dako	Thermo, 34075
Fibronectin	CHEMICON, MAB88904	Mouse	3% BSA	1/2000	1/20000, Jackson	Amersham
Collagen-I	Novus Biologicals, NB600-408	Rabbit	3% BSA	1/2000	1/20000, Jackson	Amersham
FZD8	AbCam, ab155095	Rabbit	5% BSA	1/500	1/1000, Dako	Thermo, 34075
β-Actin	AbCam, ab8227	Rabbit	5% BSA	1/1000	1/2000, Dako	Thermo, 32106
β-Actin	Abgent, AM1021B	Mouse	5% BSA	1/1000	1/2000, Dako	Thermo, 32106
